# Screening of Antibacterial Activity of Some Resupinate Fungi, Reveal *Gloeocystidiellum lojanense* sp. nov. (Russulales) against *E. coli* from Ecuador

**DOI:** 10.3390/jof9010054

**Published:** 2022-12-29

**Authors:** Andrea Jaramillo-Riofrío, Cony Decock, Juan Pablo Suárez, Ángel Benítez, Gabriel Castillo, Darío Cruz

**Affiliations:** 1 Microbial Systems Ecology and Evolution MS2E, Biodiversity of Tropical Ecosystems BIETROP Research Groups, Departamento de Ciencias de la Salud, Departamento de Ciencias Biológicas y Agropecuarias, Universidad Técnica Particular de Loja, San Cayetano Alto s/n, Loja 1101608, Ecuador; 2 Département de Biologie, Ecologie et Evolution, Université de Liège, Quai Van Beneden, BE-4000 Liège, Belgium; 3 Mycothèque de l’Université Catholique de Louvain (MUCL, BCCM), Earth and Life Institute–Microbiology (ELIM), Université Catholique de Louvain, Croix du Sud 2 bte L7.05.06, BE-1348 Louvain-la-Neuve, Belgium; 4 Instituto Nacional de Biodiversidad INABIO, Quito 17078976, Ecuador

**Keywords:** Basidiomycota, Cajanuma, Gloeocystidiellaceae, ITS1-5.8S-ITS2, resupinates

## Abstract

Bacterial resistance to antibiotics is a serious public health problem that needs new antibacterial compounds for control. Fungi, including resupinated fungi, are a potential source to discover new bioactive compounds efficient again to bacteria resistant to antibiotics. The inhibitory capacity against the bacterial species was statistically evaluated. All the species (basidiomata and strains) were molecularly characterized with the ITS1-5.8S-ITS2 barcoding marker. The strains *Ceraceomyces* sp., *Fuscoporia* sp., *Gloeocystidiellum* sp., *Oliveonia* sp., *Phanerochaete* sp., and *Xenasmatella* sp. correspond to resupinate Basidiomycetes, and only the strain *Hypocrea* sp. is an Ascomycete, suggesting contamination to the basidiome of *Tulasnella* sp. According to the antagonistic test, only the *Gloeocystidiellum* sp. strain had antibacterial activity against the bacterial species *Escherichia coli* of clinical interest. Statistically, *Gloeocystidiellum* sp. was significantly (<0.001) active against two *E. coli* pathotypes (O157:H7 and ATCC 25922). Contrarily, the antibacterial activity of fungi against other pathotypes of *E. coli* and other strains such as *Serratia* sp. was not significant. The antibacterial activity between 48 and 72 h increased according to the measurement of the inhibition halos. Because of this antibacterial activity, *Gloeocystidiellum* sp. was taxonomically studied in deep combined morphological and molecular characterization (ITS1-5.8S-ITS2; partial LSU D1/D2 of nrDNA). A new species *Gloeocystidiellum lojanense*, a resupinate and corticioid fungus from a tropical montane rainforest of southern Ecuador, with antibacterial potential against *E. coli*, is proposed to the science.

## 1. Introduction

Pathogenic microorganisms (e.g., bacteria, fungi, and viruses) that cause infections are a serious public health problem that is increasing considerably, mainly by the high rate of genetic changes, resistance mechanisms, or wrong and excessive use of antimicrobials [1,2,3]. In addition, bacterial resistance to antibiotics increases infection rates (i.e., Gram-negative bacteria, 61.3%; Gram-positive bacteria, 34.8%; yeasts, 2%; and other pathogens, 1.9%) mainly in developing countries [2,3,4,5].

The Enterobacteriaceae family is the largest and most heterogeneous group of Gram-negative bacteria of clinical importance [6]. Within this group, genera such as *Citrobacter*, *Enterobacter*, *Escherichia*, *Klebsiella*, *Proteus*, *Serratia*, *Shigella*, and *Salmonella* are the most frequent causes of human infections [7,8,9]. About 80% of infections, including urinary tract infections, pneumonia, diarrhea, meningitis, sepsis, and endotoxic shock, among others, are caused by Enterobacteriaceae [5,9,10,11,12].

On the other hand, according to the reviewed literature, an alarming decrease in the discovery of antibiotics has been observed during recent decades [13,14], which probably involves factors such as the lack of interest from the pharmaceutical industry [15], as well as the lack of resources intended for research and bioprospecting of organisms [16]. In this sense, the need arises to look for new molecules or proteins with antibacterial properties, such as synthetic or natural compounds, investigated mainly in plants and very poorly in fungi [17].

Fungi constitute a promising group of interest for the search for bioactive compounds [18], in addition to being a highly diverse group of organisms, with an estimated 1.5 to 5 million species in the world [19], of which only a small proportion of <100,000 species have been described, according to Baldrian et al. [20]. This group of organisms is able to adapt to and survive extreme conditions in several ecosystems [21]. This characteristic may be due to the production of a wide range of bioactive compounds [22]. For example, filamentous fungi, mainly Ascomycetes (e.g., *Aspergillus*, *Cladosporium, Fusarium, Penicillium notatum*), are able to produce enzymes, microbial biomass, and secondary metabolites, including antibiotics (e.g., fusidic acid, cephalosporin, and penicillin), that are applied for the treatment of various infectious diseases [18,23,24]. Likewise, Basidiomycete fungi generate a large number of metabolites that have demonstrated antibacterial, antifungal, antiviral, cytotoxic, and hallucinogenic capacities [25,26].

Among the fungi, the macro Basidiomycetes *Lentinus edodes*, followed by species within the genera *Boletus*, *Ganoderma*, and *Lepista* are promising candidates for the search for compounds with antibiotic activity against Gram-positive and Gram-negative bacteria [25]. Likewise, other fungi with resupinate and corticioid characteristics, such as *Perenniporia* spp. and *Antrodia* spp. in the order Polyporales, have been evaluated for antimicrobial activity [26]. However, there is still a low number of prospective mushroom studies, especially in neotropical areas such as Ecuador, where these organisms are still poorly cataloged [27,28,29,30], characterized morphologically and molecularly [31,32,33,34], or even more chemically characterized by evaluating the metabolites that they generate [35]. However, mushrooms with medicinal properties, from an ethnomycological point of view, have been reported in Ecuador [36,37,38,39].

Most research focused on fungal antibacterial compounds has been conducted mainly from macrofungi due to their ease of characterization and cultivation [25]. However, the study of fungi that are almost imperceptible to the naked eye, such as “resupinates”, which are distributed in several taxonomic groups within Homobasidomycetes and Heterobasidomycetes, has been left aside [40]. Resupinated fungi exhibit diverse basidiomata forms (e.g., corticoid, trechisporoid, jaapiaid, poliporoid, russuloid, Heimenoquetide, and Cantharelloides) [40] and fulfill ecological roles, such as saprotrophs (decomposing organic matter) [41,42], symbionts (forming mycorrhizae) [31,43], or parasites of insects or plants [44]. The integration of molecular, morphological [32], and biochemical [35] characterization has allowed the discovery of new species with different biotechnological potentials [45].

Therefore, this research aims to evaluate the antibacterial potential of seven fungal strains isolated from resupinate basidiomata from a tropical montane forest (Podocarpus National Park (PNP)) in southern Ecuador, against bacteria of clinical interest, such as *Escherichia coli*, *Serratia* sp., and *Klebsiella* sp. We describe *Gloeocystidiellum lojanense* as a new species to science, integrating a morphological and molecular (ITS1-5.8S-ITS2; partial LSU D1/D2 nrDNA) characterization, and we report for the first time the antibacterial activity of *G. lojanense* against the Gram-negative bacteria *Escherichia coli*.

## 2. Materials and Methods

### 2.1. Study Area

**Figure 1:**
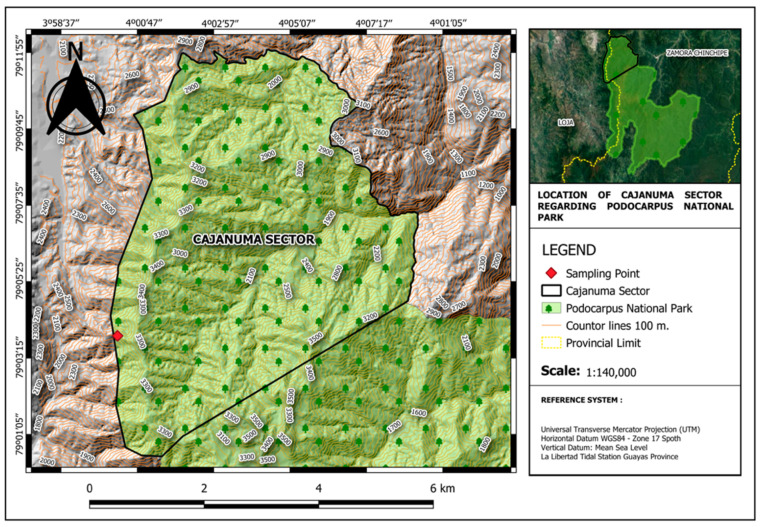
Location of sampling area in Cajanuma within the Podocarpus National Park (PNP); located in the provinces of Loja and Zamora Chinchipe, Ecuador.

Seven resupinate basidiomata were collected in the Podocarpus National Park (PNP) Cajanuma sector, Loja, Ecuador, between 2500 and 3000 m a.s.l. (Figure 1), in a tropical montane rainforest with a characteristic flora structure with dominant trees of the canopy generally exhibit twisted or tortuous trunks and branches, presenting smaller, leathery leaves [46,47,48]. The specimens are deposited in the fungarium of the Herbarium of the Universidad Técnica Particular de Loja HUTPL(F), under the codes [HUTPL(F)2162, 2166, 2181, 2183, 2187, 2198, 2203]. The pure strains isolated from the basidiomes were obtained by sporulation on two plates of potato dextrose agar (PDA) + chloramphenicol 1%. The strains were kept in the HUTPL annexed strain collection, under the codes [HUTPL(F)550, 551, 552, 553, 554, 555, 556].

### 2.2. Morphological Analysis

**Figure 2:**
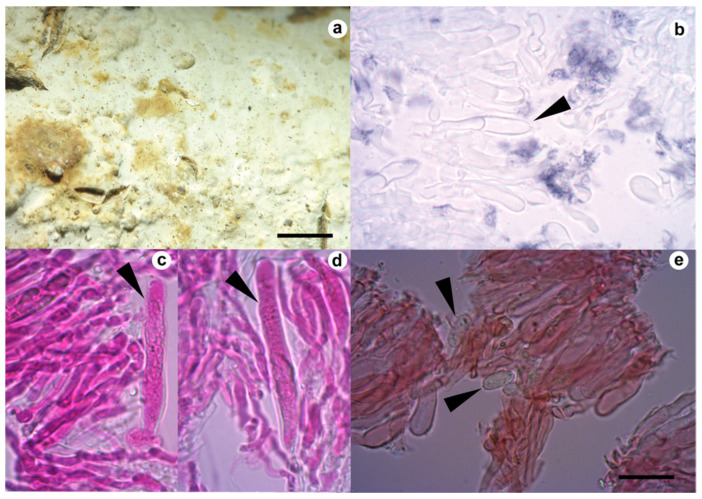
*Gloeocystidiellum lojanense*: (**a**). basidiome; (**b**). gloecystidias (black arrowhead) in Meltzer’s reagent; (**c**,**d**). gloecystidias (black arrowheads) with granular protoplasmic content in Phloxine; (**e**). basidiospores in Congo Red. Scale bar = 10 μm except (**a**) 1 cm.

**Figure 3:**
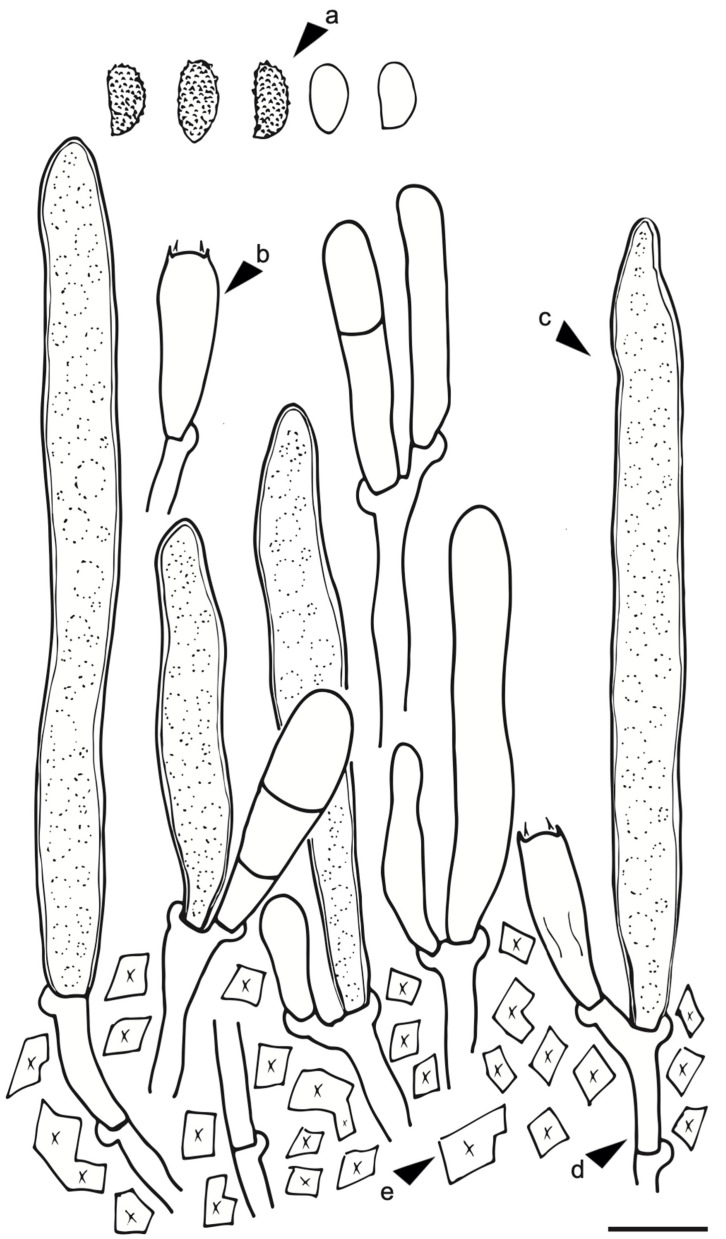
*Gloeocystidiellum lojanense* sp. nov. coll. [HUTPL(F)2181] (holotype): (**a**). basidiospores verrucose and basidiospores smooth (immature); (**b**). basidia clavate; (**c**). gloecystidia; (**d**). hyphae with clamps; (**e**). calcium oxalate. Scale bars = 10 μm.

Only *Gloeocystidiellum* sp., after positive antibacterial activity, was analyzed morphologically in detail. In order to perform the analysis, freehand sections were made with a razor blade under a stereomicroscope (Stemi Carl Zeiss). The microscopic procedure followed Xing et al. [49]. The preparations of the sections were with phloxine 1% and decolorization with 10% potassium hydroxide (KOH) solution and Congo Red 1%. The amyloid reaction was evaluated with Melzer’s reagent (Figure 2). A sulfoaldehyde (SA) reaction to detect a sulfuric reaction of gloecystidia was performed with sulfuric acid + vanillin (Sigma-Aldrich). Observations were under a light microscope (CX31, Olympus) at 100× magnification. A detailed illustration (Figure 3) of the specimen was performed by hand using a scale (1 × 1 cm^2^ = 5 × 5 µm^2^) and later revision of the taxonomic key for the genus available in Wu, Larsson, and Ryvarden [50,51]. Color codes are based on the online server https://encycolorpedia.es/ (11 July 2022) [52].

### 2.3. Molecular Analysis

DNA was isolated from fresh basidiomata using the Phire Plant Direct Master Mix PCR Kit (Thermo Scientific™) and subsequently from pure fungal strains with the InnuPREP DNA Kit (Analytik-jena™) according to the manufacturer’s instructions. For the polymerase chain reaction (PCR), the ITS1 (5′CCGTAGGTGAACCTGCGG3′) and NL4 (5′GGTCCGTGTTTCAAGACGG3′) primers were used [53] to amplify the internal transcribed spacer (ITS) region and a partial sequence of nuclear large subunits (LSUs). The reactions were carried out under the following conditions: initial denaturation (98 °C, 5 min), followed by 40 cycles with denaturation (98 °C, 10 s), hybridization (55 °C, 10 s), extension (72 °C, 30 s), and final extension (72 °C, 5 min). Final reaction volume was 20 µL, including 1 µL of the extracted DNA for each reaction. The PCR product was purified with the PureLink™ PCR Purification Kit (Thermo Scientific™) and sequenced at Macrogen Inc. (Seoul, Korea), with the same set of primers used for PCR amplification. All sequences corresponding to the basidiomata, and strains were subjected to a BLAST search against the GenBank database (https://www.ncbi.nlm.nih.gov) as well as the UNITE database (https://unite.ut.ee).

Only the sequences corresponding to *Gloeocystidiellum* sp. were assembled and edited using Lasergene 7 (DNAStar, Madison, WI, USA). The sequences obtained in this study were submitted to the NCBI nucleotide database under the accession numbers presented in Table 1. The alignment of the sequences obtained and reference sequences downloaded from GenBank (Table 1) was performed in the program MAFFT 7 [54] under the GINSI algorithm. Alignments were visualized in PhyDE software [55] in order to check for ambiguities, especially at the tails, for a manual adjustment if required. The alignments were analyzed by means of a neighbor-joining (NJ) approach using a Kimura two-parameter (K2P) with 1000 bootstrap repetitions (BS) and a maximum likelihood using the general time-reversible (GTR) method with 1000 bootstrap repetitions. Both analyses were performed with MEGA 11 software [56].

**Figure 4:**
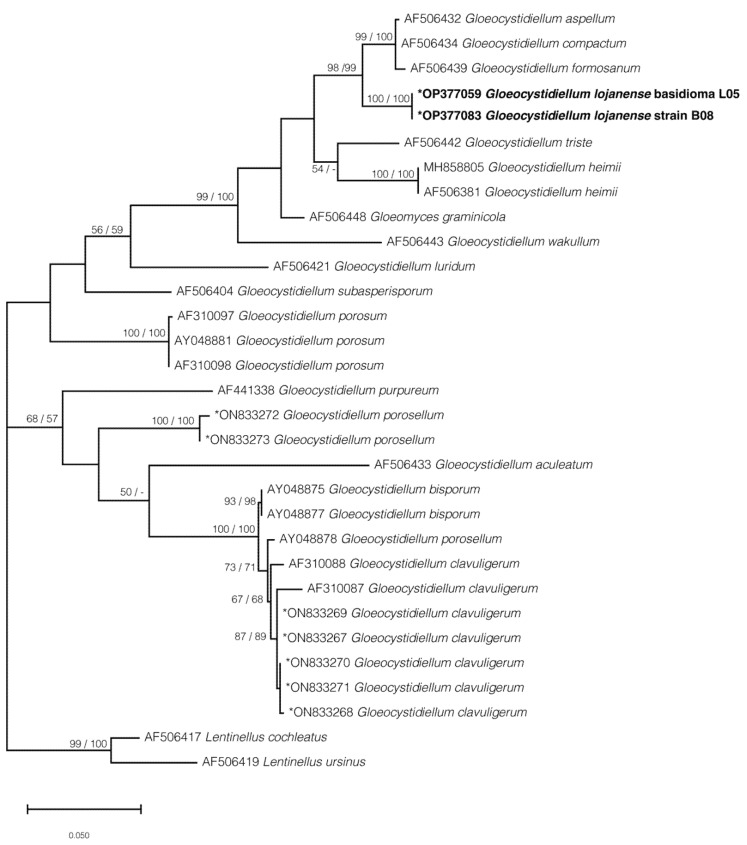
Concatenated maximum likelihood phylogenetic tree (ITS1-5.8S-ITS2 and partial LSU D1/D2) for sequence positioning corresponding to *Gloeocystidiellum lojanense* sp. nov. Values on the nodes correspond to ML and NJ, respectively. Bar = number of substitutions expected per position. * Sequences generated in this study. Sequences and accession numbers of the new species are in bold.

**Figure 5:**
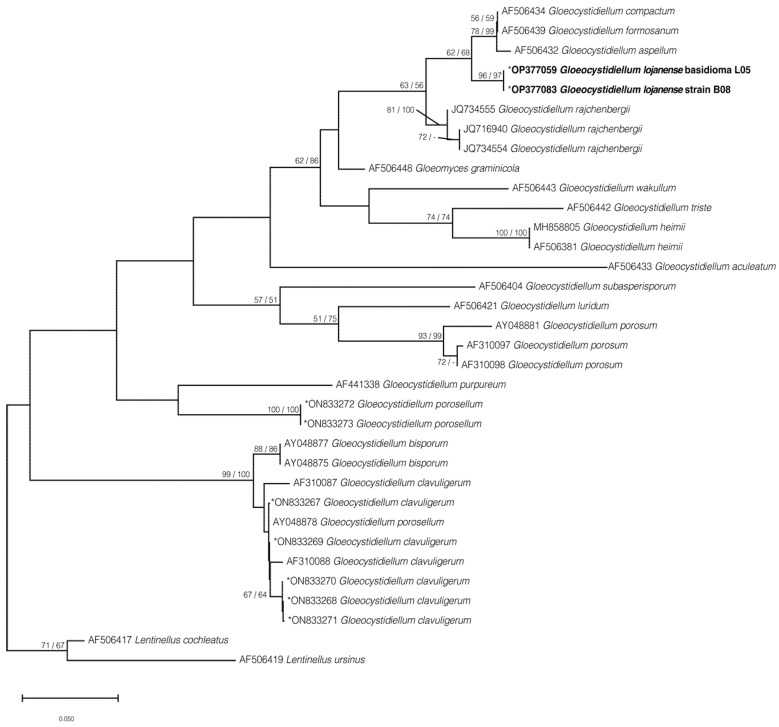
Maximum likelihood phylogenetic tree for the ITS1 region for sequence positioning corresponding to *Gloeocystidiellum lojanense* sp. nov. and positioning of *Gloeocystidiellum rajchenbergii*. Values on the nodes correspond to ML and NJ, respectively. Bar = number of expected substitutions per position. * Sequences generated in this study. Sequences and accession numbers of the new species are in bold.

Finally, four phylogenetic trees were calculated: the first includes 28 sequences for the region (ITS1-5.8S-ITS2) (Figure S1), the second tree includes 36 sequences for the partial LSU region (D1/D2) (Figure S2), the third tree corresponds to 31 concatenated sequences (ITS1-5.8S-ITS2 and partial LSU D1/D2) (Figure 4), and the fourth tree includes 34 sequences that correspond only to the ITS1 region (Figure 5) due to the requirement of comparing three short sequences (JQ7345551, JQ7169401, JQ7345541) described in Chile by Gorjón and Hallenberg [57]. All the phylogenetic trees presented in this study were inferred using a maximum likelihood approach.

Interspecific variation between sequences from specimen HUTPL(F)2181 and close phylogenetic species was evaluated by pairwise distances for the ITS1-5.8S-ITS2 regions and partial LSU D1/D2 in the MEGA 11 software with a Kimura two-parameter distance [58] by the “partial deletion” and “complete deletion” method.

Additionally, sequences from seven strains of various *Gloeocystidiellum* spp. (MUCL33964, MUCL33965, MUCL33966, MUCL33967, MUCL33968, MUCL35247, MUCL35248) were included in the phylogenetic analyses. These strains are maintained in the collection of the Mycothèque de l’Université catholique de Louvain (MUCL/BCCM), Louvain-la-Neuve, Belgium.

### 2.4. Antibacterial Activity

#### 2.4.1. Tested Bacterias

Ten Gram-negative bacterial strains of clinical interest were used in this study to evaluate the antibacterial activity of seven strains from resupinated fungi. These strains correspond to six different species: *Enterobacter aerogenes*; *Enterobacter cloacae*; *Escherichia coli*, including two pathotypes (uropathogenic *Escherichia coli* (UPEC), phylogenetic group (GF) A; GF B2; O157:H7) and the certified strain *E. coli* ATCC 25922 GF: B2; *Klebsiella pneumoniae* (ATCC BAA-1706); *Pseudomonas aeruginosa*; and *Serratia* sp. All the strains are available in the strain collection of the Laboratory of Cultures and Conservation of Microorganisms–UTPL, and were previously characterized with standard biochemical tests and API20E tests.

#### 2.4.2. Antibacterial Activity Assay

Antibacterial activity was determined using the agar disc diffusion method by the Kirby–Bauer method as described in CLSI M100 [59]. All bacterial strains were grown on Trypticase Soy Agar (TSA, DIFCO) for 24 h at 37 °C. Subcultured colonies were suspended in 0.85% saline, followed by 0.5 McFarland density adjustment (1.5 × 10^8^ CFU/mL) by spectrophotometry.

The antibacterial tests were carried out by antagonism in a Petri dish (9 cm diameter) with Mueller–Hinton agar. Discs (0.5 cm diameter) of PDA medium colonized with the strains replaced similar space (three replicates) in the medium with bacterial growth. All plates were prepared and incubated at 37 °C for 24–72 h. Antibacterial activity was recorded as diameter (mm) of the zone of inhibition formed around the fungal disc (Figure S1). Commercial antibiotic discs for susceptibility testing based on CLSI reports [59,60,61] were also used as positive controls. Antibiotics correspond to 30 µg of cefepime for all strains, except for *K. pneumoniae* and uropathogenic *E. coli* phylogenetic group B2, in which 10 µg of imipenem was used as a positive control due to its bacterial resistance. Each Petri dish with bacterial growth is considered as one test, carried out in triplicate.

### 2.5. Data Analysis

Statistical analysis was performed by calculating the average ($\bar{X}$) of the three repetitions of each test. Prior to the analysis, the dependent variable “inhibition” was transformed to a logarithm in base 2 to fulfill the assumption of normality (*p* > 0.05). Two-way analysis of variance to test the effects of *Gloeocystidiellum* sp. strain (the only fungus with antibacterial activity) and event on bacterial inhibition was used. Finally, a post-hoc Tukey test to check differences in inhibition between pairs of bacteria was performed. All analyses were carried out in R Studio software (RStudio, Inc., Boston, MA, USA) [62].

## 3. Results

Six of seven strains identify as five Basidiomycetes, and one ascomycete under six orders (Table 2) was not characterized taxonomically because they did not present antibacterial activity. On the other hand, *Gloeocystidiellum* sp., which presents positive antibacterial activity against *E*. *coli*, is taxonomically described as a new species for science. The BLAST identity percent found between 87 and 97 suggests a possibility to find some new species for the science in this tropical forest.

### 3.1. Taxonomy

*Gloeocystidiellum lojanense* A. Jaramillo, D. Cruz & C. Decock., sp. nov. (Figure 2 and Figure 3).

ETYMOLOGY: The specific epithet refers to Loja province (Ecuador) where the species was found for the first time.

BASIDIOMA—resupinate, ceraceous, or subceraceous. Hymenial surface is bright grayish white (#ecf0d4) and slightly light yellow (#b0ac6e), extending smoothly and slightly tuberculate over the substrate up to about 15 cm^2^; bounded margins.

HOLOTYPE—South America, Ecuador, Loja Province, and Canton, Podocarpus National Park, Cajanuma sector, alt. c. 2900 m, on fallen decomposing branch of unknown tree, 23 February 2021. A. Jaramillo [HUTPL(F)2181].

Microscopic Structure. Hyphal system monomitic: generative hyphae 3–4 µm diameter, septate, thin- to thick-walled hyaline with clamp connection with calcium oxalate crystals, gloeocystidia abundant, tubular or cylindrical, slightly tapering towards the apices, thick-walled throughout, up to approximately 80–90 μm long, 7–8 μm wide, the protoplasmic content granular and yellowish in KOH, blackish in SA (positive reaction), basidia clavate 25–35 long × 5–6 μm wide, transversely septate at the basal zone, with four sterigmata, basidiospores hyaline ellipsoid and slightly verrucose, thin-walled, 6.5–8 μm long × 3.4–4.5 μm wide, and slightly amyloid under Meltzer’s reagent (Figure 2 and Figure 3).

*Gloeocystidiellum lojanense* is morphologically related to *Gloeocystidiellum formosanum*, *G. compactum*, *G. aspellum*, and *G. rajchenbergii* [50,57]. These species are closely similar as far as their morphology is concerned, especially the basidiospores and gloeocystidia features (Table 3); additionally, all of them present an amyloid reaction in Meltzer’s reagent. Microscopically, the shape and size (Table 3) of basidiospores and gloeocystidia in *G. lojanense* are indistinguishable from those of its allied species listed above. Although the size of the basidiospores has been considered as the main characteristic to define species in this group [50,57,63,64,65], it is evident that they can overlap each other, causing taxonomic confusion, as has already been indicated in other groups of fungi (e.g., *Tulasnella* spp.), where molecular data allowed revealing some cryptic morphological species [32].

Another reason to propose *G. lojanense* as a new neotropical species is their biogeographic and ecological location, as has been described in other *Gloeocystidiellum* species [64,65]. *G. lojanense* was found in a tropical montane rainforest in southern Ecuador at 2900 m asl; different to the places in eastern Asia (Taiwan, China) or Patagonia Andean of Chile, about 600 to 1250 m asl, reported to the closest morphological species [50,57,66].

### 3.2. Phylogenetic Hypothesis for Gloeocystidiellum lojanense

The phylogenetic results, based on the ITS or the concatenated data sets (Figure 4 and Figure 5; Figures S2 and S3) showed that *G. lojanense* form an independent well-supported clade (respectively, 96 and 100% bootstrap) that is here interpreted as a new phylogenetic species. *Gloeocystidiellum lojanense* forms a sister clade (Figure 5, BS 98/99% for ML/NJ) to the clade gathering *G. aspellum*, *G. compactum*, and G. *formosatum* from several countries (Table 1). A similar topology if observed is when the ITS1 region (63/56% BS for ML/NJ), including the sequence from the species *G. rajchenbergii*, is analyzed.

The phylogenetic tree obtained from the concatenated dataset (ITS1-5.8S-ITS2 and partial LSU D1/D2 regions) (Figure 4) is consistent with the phylogenies from independent analyses for the ITS1 (Figure 5) or ITS1-5.8S-ITS2 (341 bp) regions (Figure S1) and LSU D1/D2 partial (471 bp) (Figure S3), where *G. lojanense* is grouped as a sister group with the species *G. aspellum*, *G. compactum*, *G. formosatum*, and *G. rajchenbergii* but remains as an independent clade with BS values higher than 95/97% for ML/NJ.

The resulting interspecific groups are totally supported when the sequences were analyzed by complete deletion and partial deletion; for example, the range for the ITS1-5.8-ITS2 region is between 6.45% and 6.90% (Table 4), ITS1 is between 2.94 and 3.69%, and partial LSU D1/D2 is 0.81% (Table 4).

### 3.3. Antibacterial Activity

*Gloeocystidiellum lojanense* HUTPL(F)550 was the unique strains tested by inhibiting the bacterial strains of *Escherichia coli* and *Serratia* sp. (Table 5). The other six strains (Table 2) were not further charactrized because they were totally inactive against the bacterias tested.

**Figure 6:**
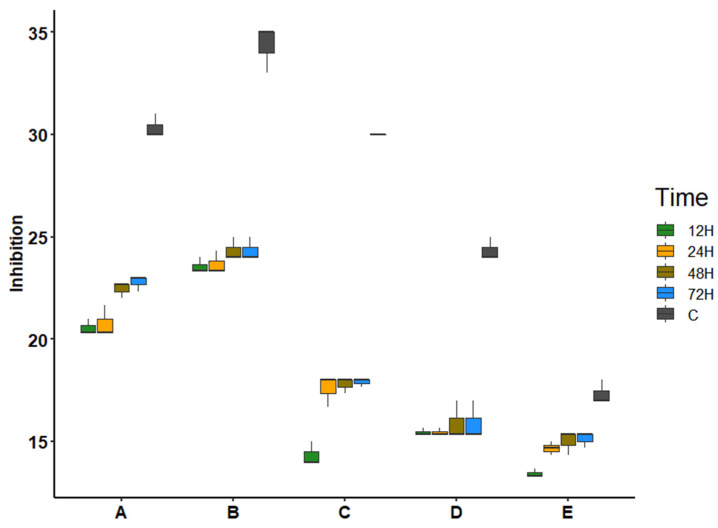
Box plot of the effects of bacteria and time on inhibition: (**A**): *Escherichia coli* ATCC 25922; (**B**): *Escherichia coli* O157:H7; (**C**): *Escherichia coli* UPEC phylogroup A; (**D**): *Escherichia coli* UPEC phylogroup B2; (**E**): *Serratia* sp.

*Gloeocystidiellum lojanense* was observed to be more effective at inhibiting mainly two pathotypes of *E. coli* (i.e., *Escherichia coli* ATCC 25922 and *Escherichia coli* O157:H7). The inhibition halos generated for these strains were larger in size (between 22.78 and 24.33 mm in diameter (Table 5) and statistically significant (<0.001) versus the other tests (Figure 6, Table 6). However, the inhibition halos generated by *G. lojanense* did not exceed the halos generated from the positive controls (30.33–34.33 mm in diameter) for the strains *Escherichia coli* ATCC 25922 and *Escherichia coli* O157: H7. Inhibition halos varied according to the incubation time (Table 5).

The bacteria *Enterobacter aerogenes*, *Enterobacter cloacae*, *Klebsiella pneumoniae*, *Klebsiella pneumoniae* ATCC BAA-1706, and *Pseudomonas aeruginosa* did not exhibit inhibitory activity by *G. lojanense*.

The box plot clearly indicates that *Gloeocystidiellum lojanense* presents greater inhibition in the bacteria *Escherichia coli* ATCC 25922 (22.44/22.78 mm) and *Escherichia coli* O157:H7 (24.33 mm), increasing during 48 and 72 hours, compared to *Escherichia coli*. UPEC FG: A, *Escherichia coli* UPEC FG: B2 and Eb, EGA, and *Serratia* sp., which were more stable over time (Figure 6).

The analysis of variance indicated that the fungal species, time, as well as their interaction between the two factors, significantly affect the inhibition of the bacteria, showing significant values of each factor (<0.001) (Table 6).

## 4. Discussion

*Gloeocystidiellum lojanense* represents a species new to science from a tropical montane rainforest in southern Ecuador. This species is phylogenetically supported, forming an independent clade with BS values greater than 96% for the ITS1-5.8S-ITS2 and partial LSU regions, analyzed independently or concatenated. This species presents an interspecific genetic divergence for the ITS1-5.8S-ITS2 region greater than 6.45% with respect to the other species. This interspecific divergence of 6.45% would generate a barcode gap with respect to the 3% or 4% thresholds of intraspecific variability studied for fungi [32,67].

Morphologically and molecularly, *G. lojanense* is suggested as a sister group to the clade comprising *G. formosanum*, *G. compactum*, *G. aspellum*, and *G. rajchenbergii* [57]. All these species are practically indistinguishable morphologically, mainly due to the overlap of shapes and sizes of basidiospores and gloeocystidia. This problem, discussed as “cryptic species”, is already well known to other groups such as *Tulasnella* spp. [32], where several morphologically cryptic species were delimited genetically. Other species (i.e., *Gloeocystidiellum clavuligerum* and *G porosum*) are morphologically [63,64,65] closely related to *G. lojanense* but genetically distant (>6.45% ITS1-5.8S-ITS2), forming a different clade (Figure 4). Additionally, the interspecific genetic difference for the ITS1-5.8S-ITS2 region is greater than 6.45%, exceeding the so-called barcode gap discussed by [32].

*Gloeocystidiellum lojanense* exhibited antibacterial activity against the four strains of the *E. coli* species, with *E. coli* ATCC 25922 and *E. coli* O157:H7 (Figure 6) showing greater inhibition than the other *E. coli* strains. This inhibitory action may be due to the fact that these *E. coli* pathotypes are less virulent and resistant [68]. Other pathotypes (e.g., *Escherichia coli* UPEC FG: A, *Escherichia coli* UPEC FG: B2) are reported to be resistant to antibiotics [69], observed in slight size changes in the inhibition halos. Likewise, *G. lojanense* did not significantly inhibit the growth of *Serratia* sp. This species is also known to have a high bacterial resistance to antibiotics [70], as evidenced in the positive control with a smaller size of the inhibition halo. Suay et al. [71] present a study indicating that the species *Gloeocystidiellum porosum* has antibacterial activity against *Pseudomonas aeruginosa*, *Serratia marcescens*, and *Staphylococcus aureus*, which is why a further exploration of this group of fungi is required.

Antibacterial inhibition increased between 48 and 72 h, probably because the fungal metabolites exhibiting these antibacterial properties were generated secondarily by nutrient depletion, or simply by recognition of the bacterial foreign agent [72]. Six different strains of resupinated fungi within (Amylocorticiales, Cantharellales, Hymenochaetales, Polyporales, Russulales) and one *Hypocrea* sp. (Hypocreales) did not exhibit inhibition activity against any bacterial strain tested. The *Gloeocystidiellum* sp. (active against bacteria) and *Xenasmatella* sp. (inactive against bacteria) share the same order Russulales but differ in antibacterial activity. Many studies report different fungi species in the same order such as this study (e.g., Polyporales, Russulales) but positive for bacteria inhibitions [25,26]. Probably, the antibacterial activity of fungi is restricted at the species level by specific genetic adaptations [73] requiring further analysis of the gene expression of these fungi.

Statistically, the antibacterial activity of *G. lojanense* is significant mainly for two pathotypes of *E. coli* (i.e., *E. coli* ATCC 25922 and *E. coli* O157:H7). However, our analysis requires an evaluation of the minimum inhibitory concentration by obtaining its extracts, as has already been evaluated for other fungi [74].

Ecuador, considered within the megadiverse countries worldwide [75,76,77], has as consequence a great diversity of fungi [27,29,33,34] that require integrative taxonomic research to discover new species such as *G. lojanense* with bioactive potential and applications in several sectors such as human health.

## Figures and Tables

**Table 1:** GenBank sequences reference applied in molecular analyses in this study.

Taxon	Herbarium/Collection	Provenance	GenBank	Sequence Region
*Gloeocystidiellum aculeatum*	GB/2647	Taiwan	AF265546	ITS, nuLSU
*Gloeocystidiellum aculeatum* *	GB/2647	Taiwan	AF506433	ITS, nuLSU
*Gloeocystidiellum aspellum* *	GB/LIN625	Taiwan	AF506432	ITS, nuLSU
*Gloeocystidiellum aspellum*	HE4262	China	KY860460	nuLSU
*Gloeocystidiellum aspellum*	HE2041	China	KY860461	nuLSU
*Gloeocystidiellum aspellum*	HE1773	China	KY860462	nuLSU
*Gloeocystidiellum bisporum*	CBS/961.96	Sweden	AY048875	ITS, nuLSU
*Gloeocystidiellum bisporum*	GB/KHL4700	Sweden	AY048876	ITS
*Gloeocystidiellum bisporum*	GB/KHL11135	Norway	AY048877	ITS, nuLSU
*Gloeocystidiellum clavuligerum*	HHB1046	USA, Mich.	AF310087	ITS, nuLSU
*Gloeocystidiellum clavuligerum*	GB/NH11185	Spain, Tenerife	AF310088	ITS, nuLSU
*Gloeocystidiellum clavuligerum*	HE3313	China	KY860441	nuLSU
*Gloeocystidiellum clavuligerum*	MUCL/33964	France	**ON833267**	ITS, nuLSU
*Gloeocystidiellum clavuligerum*	MUCL/33965	France	**ON833268**	ITS, nuLSU
*Gloeocystidiellum clavuligerum*	MUCL/33966	France	**ON833269**	ITS, nuLSU
*Gloeocystidiellum clavuligerum*	MUCL/33967	France	**ON833270**	ITS, nuLSU
*Gloeocystidiellum clavuligerum*	MUCL/33968	France	**ON833271**	ITS, nuLSU
*Gloeocystidiellum compactum*	NMNS/WU880615-21	Taiwan	AF506434	ITS, nuLSU
*Gloeocystidiellum formosanum*	NMNS/WU9404-19	Taiwan	AF506439	ITS, nuLSU
*Gloeocystidiellum friesii*	CBS/323.66	France	MH870446	nuLSU
*Gloeocystidiellum graminicola* *	GB/WU9210-12	Taiwan	AF506448	ITS, nuLSU
*Gloeocystidiellum heimii*	LY/CBS321.66	C. African Rep.	AF506381	ITS, nuLSU
*Gloeocystidiellum heimii*	LY/CBS321.66	C. African Rep.	MH858805	ITS, nuLSU
*Gloeocystidiellum kenyense*	TFC/15278	Portugal	FR878082	ITS
*Gloeocystidiellum kenyense*	TFC/15309	Portugal	FR878083	ITS
*Gloeocystidiellum kenyense*	MA/Fungi80408	Portugal	FR878084	ITS
*Gloeocystidiellum leucoxantha*	CBS/454.86	USA	AF287860	nuLSU
*Gloeocystidiellum lojanense* *	HUTPL(F)/2181	Ecuador	**OP377059**	ITS, nuLSU
*Gloeocystidiellum lojanense*	HUTPL(F)/550	Ecuador	**OP377083**	ITS, nuLSU
*Gloeocystidiellum luridum*	HK9808	Sweden	AF506421	ITS, nuLSU
*Gloeocystidiellum porosellum*	HJM8851	Sweden	AY048878	ITS, nuLSU
*Gloeocystidiellum porosellum*	MUCL/35247	France	**ON833272**	ITS, nuLSU
*Gloeocystidiellum porosellum*	MUCL/35248	France	**ON833273**	ITS, nuLSU
*Gloeocystidiellum porosum*	CBS/51085	Netherlands	AF310097	ITS, nuLSU
*Gloeocystidiellum porosum*	CBS/27154	France	AF310098	ITS, nuLSU
*Gloeocystidiellum porosum*	GB/EB990923	Sweden	AY048881	ITS, nuLSU
*Gloeocystidiellum porosum*	WU1608-176	Taiwan	LC430908	nuLSU
*Gloeocystidiellum purpurem* *	GB/WU9310-45	Sweden	AF441338	ITS, nuLSU
*Gloeocystidiellum rajchenbergii* *	GB/NH16358	Chile	JQ716940	ITS1
*Gloeocystidiellum rajchenbergii*	GB/NH16348	Chile	JQ734554	ITS1
*Gloeocystidiellum rajchenbergii*	GB/NH16353	Chile	JQ734555	ITS1
*Gloeocystidiellum subasperisporum*	GB/KHL8695	Norway	AF506404	ITS, nuLSU
*Gloeocystidiellum triste*	GB/KHL10334	Puerto Rico	AF506442	ITS, nuLSU
*Gloeocystidiellum wakullum*	O/OSLO-930107	Tanzania	AF506443	ITS, nuLSU
*Lentinellus cochleatus*	GB/KGN96-09-28	Sweden	AF506417	ITS, nuLSU
*Lentinellus ursinus*	GB/EL73-97	USA, N.C	AF506419	ITS, nuLSU

* Holotypes. Acronyms correspond to: CBS, Centraalbureau voor Schimmelcultures; GB, Herbarium University Gothenburg; HHB, H. H. Burdsall; HK, H. Knudsen; HUTPL(F), Herbario (Fungario) UTPL; LY, Université Claude Bernard Lyon 1; NMNS, National Museum of Natural Science; MA, Real Jardín Botánico; MUCL, Mycothèque UCLouvain; O, Oslo University; TFC, Universidad de La Laguna. Accession numbers to new sequences obtained in this study are in bold.

**Table 2:** BLAST similarity of basidiomata and fungal strains analyzed in this study.

Code	Source	Closest Identified Relative	Query Cover (%)	Identity (%)	Culture From Fructification	Order	Closest Identified Relative	Query Cover (%)	Identity (%)
HUTPL(F)2181	Basidiome	*Gloeocystidiellum* sp.	93	96	HUTPL(F)550	Russulales	*Gloeocystidiellum* sp.	94	95.93
HUTPL(F)2162	Basidiome	*Tulasnella* sp.	98	95.53	HUTPL(F)551	Hypocreales	*Hypocrea* sp.	98	97
HUTPL(F)2166	Basidiome	*Oliveonia* sp.	99	94.2	HUTPL(F)552	Cantharellales	*Oliveonia* sp.	97	93.79
HUTPL(F)2183	Basidiome	*Phanerochaete* sp.	99	98.11	HUTPL(F)553	Polyporales	*Phanerochaete* sp.	98	97.93
HUTPL(F)2187	Basidiome	*Ceraceomyces* sp.	90	89.1	HUTPL(F)554	Amylocorticiales	*Ceraceomyces* sp.	89	88.9
HUTPL(F)2198	Basidiome	*Fuscoporia* sp.	97	90.12	HUTPL(F)555	Hymenochaetales	*Fuscoporia* sp.	97	89.57
HUTPL(F)2203	Basidiome	*Xenasmatella* sp.	96	97.21	HUTPL(F)556	Russulales	*Xenasmatella* sp.	94	92.75

**Table 3:** Comparison of size measurements between representative structures of *Gloeocystidiellum lojanense* versus the most morphologically related species into the genus *Gloeocystidiellum*, according the data from Donk [50].

Species	Basidiospores (L)	Basidiospores (W)	Gloeocystidia (L)	Gloecystidia (W)	Basidia (L)	Basidia (W)
*Gloeocystidiellum rajchenbergii*	6–7	3–3.5	40–70	5.5–7.5	15–20	5–5.5
*Gloeocystidiellum formosanum*	6.5–7.5	2.8–3.2	100	6–12	16–22	5–6.5
*Gloeocystidiellum compactum*	5.8–6.5	3–3.4	100	5–10	17–27	5–6
*Gloeocystidiellum aspellum*	7–8	3.5–4	150	5–10	25–35	6–7
***Gloeocystidiellum lojanense***	**6.5–8**	**3.4–4.5**	**80–90**	**7–8**	**25–35**	**5–6**

Note: L: long, W: wide. The measurements are expressed in µm. Data obtained from the new species in this study are in bold.

**Table 4:** Interspecific variation percentages between *Gloeocystidiellum lojanense* and phylogenetically sister species.

Species	*Gloeocystidiellum lojanense*		
	ITS1	ITS1-5.8S-ITS2	Partial LSU (D1/D2)
*Gloeocystidiellum aspellum*	3.69/3.56%	6.90/7.02%	0.81/0.78%
*Gloeocystidiellum compactum*	2.94/2.81%	6.45/6.59%	0.81/0.78%
*Gloeocystidiellum formosanum*	2.94/2.81%	6.45/6.59%	0.81/0.78%
*Gloeocystidiellum rajchenbergii*	2.94/2.81%	-	-
*Other species*	6.78–21.16/6.39–21.77%	8.72–33.23/9.45–28.19%	1.36–5.05/1.85–5.75%

Note: Genetic difference values correspond to complete deletion and (/) partial deletion analysis parameters, respectively. (-) Unpublished data.

**Table 5:** Antibacterial activity per diameter (mm) of inhibition halos generated by *Gloeocystidiellum lojanense* against the bacterial strains under study.

	Inhibition Zone Diameter (mm)				
	Time (h)				
Pathogenic Bacteria	C	12	24	48	72
*Enterobacter aerogenes*	25.67 ± 0.58	-	-	-	-
*Enterobacter cloacae*	26.00 ± 0.00	-	-	-	-
***Escherichia coli*** **ATCC 25922 *FG*: B2**	30.33 ± 0.58	**20.56 ± 0.38**	**20.78 ± 0.77**	**22.44 ± 0.38**	**22.78 ± 0.38**
***Escherichia coli*** **O157:H7**	34.33 ± 1.15	**23.56 ± 0.38**	**23.67 ± 0.58**	**24.33 ± 0.58**	**24.33 ± 0.58**
*Escherichia coli* UPEC *FG*: A	30.00 ± 0.00	14.33 ± 0.58	17.56 ± 0.77	17.78 ± 0.38	17.89 ± 0.19
*Escherichia coli* UPEC *FG*: B2	24.33 ± 0.58	15.44 ± 0.19	15.44 ± 0.19	15.89 ± 0.96	15.89 ± 0.96
*Klebsiella pneumoniae*	21.00 ± 0.00	-	-	-	-
*Klebsiella pneumoniae* ATCC BAA-1706	20.00 ± 0.00	-	-	-	-
*Pseudomonas aeruginosa*	28.67 ± 0.58	-	-	-	-
*Serratia* sp.	17.33 ± 0.58	13.44 ± 0.19	14.67 ± 0.33	15.00 ± 0.58	15.11 ± 0.38

The symbol “-” = without activity; C = positive control; SD standard deviation is indicated by ± (*n* = 9).

**Table 6:** Two-factor analysis of variance results.

Factors	df	SS	MS	F	*p*-Value
*G. lojanense*	4	2.3629	0.5907	836	<0.001
Time	4	1.5226	0.3807	538.7	<0.001
*G. lojanense*: Time	16	0.2284	0.0143	20.2	<0.001
Residuals	50	0.0353	0.0007		

df: degrees of freedom, ss: sum of squares, ms: mean squares, f: f statistic.

## Data Availability

All data are included in this document.

## Supplementary Materials

The following supporting information can be downloaded at: https://www.mdpi.com/article/10.3390/jof9010054/s1, Figure S1: Antibacterial activity assay flowchart; Figure S2: Maximum Likelihood phylogenetic tree for the ITS1-5.8-ITS2 region for sequence positioning corresponding to *Gloeocystidiellum lojanense* sp. nov. Bar = number of expected substitutions per position. * Sequences generated in this study; Figure S3: Maximum likelihood phylogenetic tree for the LSU region for sequence positioning corresponding to *Gloeocystidiellum lojanense* sp. nov. Bar = number of expected substitutions per position. * Sequences generated in this study.
