# Supplementary material for: Screening of Antibacterial Activity of Some Resupinate Fungi, Reveal Gloeocystidiellum lojanense sp. nov. (Russulales) against E. coli from Ecuador
Source: J Fungi (Basel). 2022 Dec 29;9(1):54. doi: 10.3390/jof9010054 (PMC9867327; doi:10.3390/jof9010054)
Supplement: Supplementary file 1 [file jof-09-00054-s001.zip › Supplementary_Figure_S3.pdf]

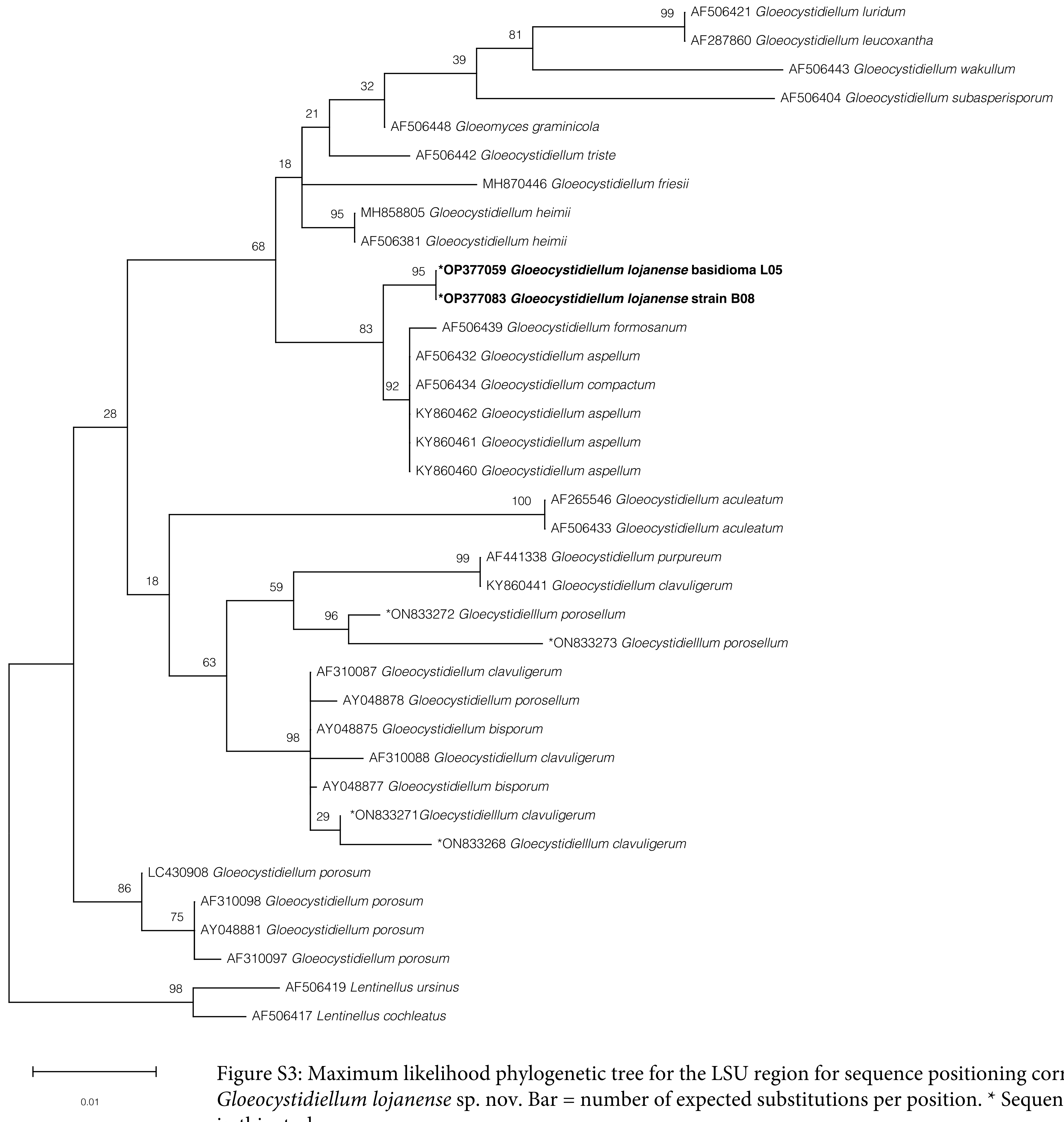

Figure S3: Maximum likelihood phylogenetic tree for the LSU region for sequence positioning corresponding to *Gloeocystidiellum lojanense* sp. nov. Bar = number of expected substitutions per position. * Sequences generated in this study.
