# Supplementary figures and images for: Screening of Antibacterial Activity of Some Resupinate Fungi, Reveal Gloeocystidiellum lojanense sp. nov. (Russulales) against E. coli from Ecuador
Source: J Fungi (Basel). 2022 Dec 29;9(1):54. doi: 10.3390/jof9010054 (PMC9867327; doi:10.3390/jof9010054)

# Antibacterial activity assay

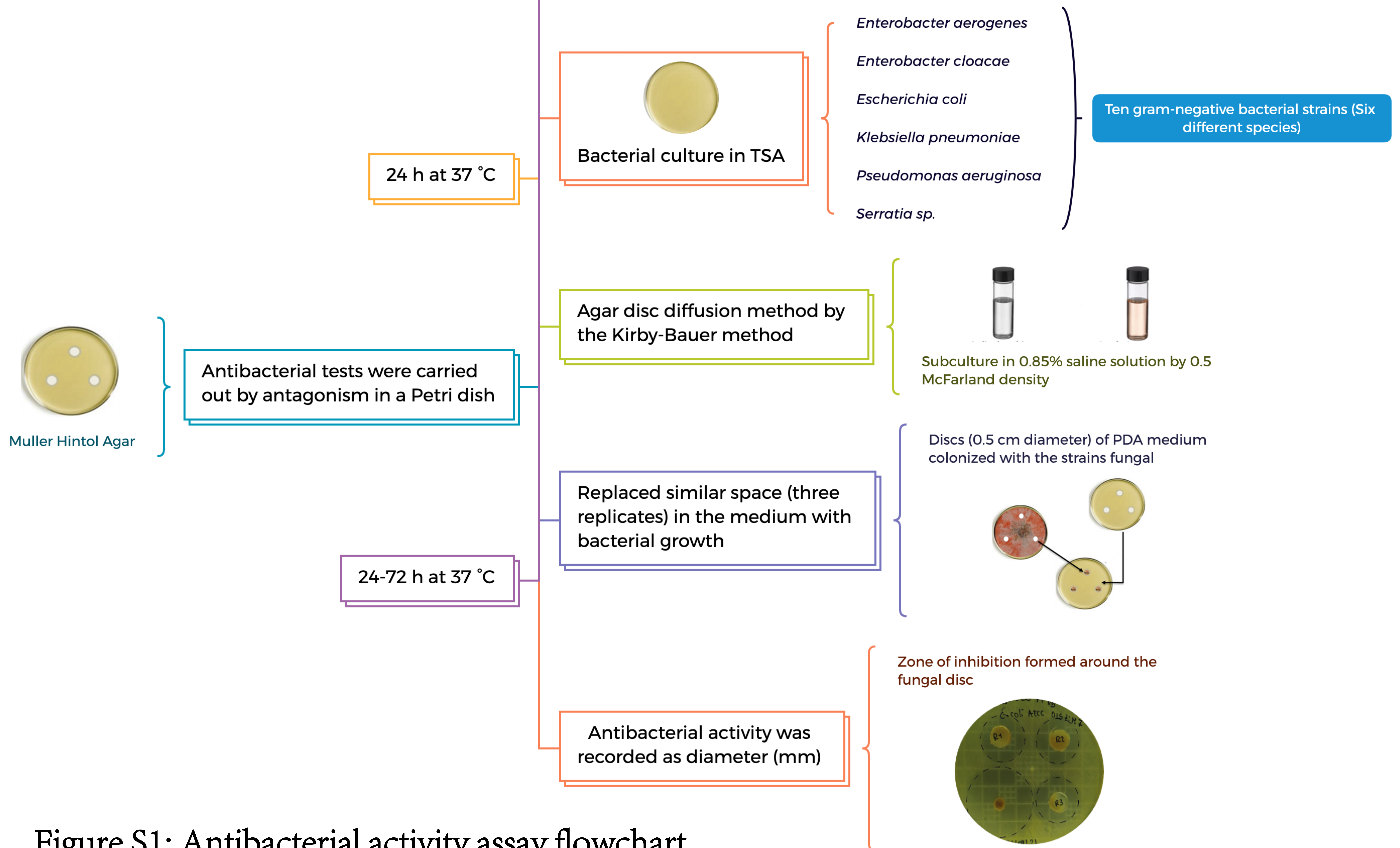

Figure S1: Antibacterial activity assay flowchart.

Supplement: Supplementary file 1 [file jof-09-00054-s001.zip › Supplementary_Figure_S1.pdf]
